# Issues Regarding the Implementation of eHealth: Preparing for Future Influenza Pandemics

**DOI:** 10.2196/ijmr.2357

**Published:** 2012-12-06

**Authors:** Junhua Li, Holly Seale, Pradeep Ray, William Rawlinson, Lundy Lewis, C. Raina MacIntyre

**Affiliations:** 1 Asia-Pacific Ubiquitous Healthcare Research CentreThe University of New South WalesSydneyAustralia; 2 School of Public Health and Community MedicineFaculty of MedicineUniversity of New South WalesSydneyAustralia; 3 School of Information Systems, Technology and ManagementAustralian School of BusinessUniversity of New South WalesSydneyAustralia; 4 Virology DivisionDepartment of MicrobiologySouth Eastern Sydney and Illawarra Health ServiceSydneyAustralia; 5 School of Medical Sciences/School of Biotechnology and Biomolecular SciencesFaculty of MedicineUniversity of New South WalesSydneyAustralia; 6 Information Technology DepartmentSouthern New Hampshire UniversityManchester, NHUnited States; 7 National Centre for Immunization Research and Surveillance of Vaccine Preventable Diseases (NCIRS)SydneyAustralia

**Keywords:** eHealth, influenza pandemic, preparedness assessment, case study

## Abstract

**Background:**

eHealth is a tool that may be used to facilitate responses to influenza pandemics. Prior to implementation of eHealth in the hospital setting, assessment of the organizational preparedness is an important step in the planning process. Including this step may increase the chance of implementation success.

**Objective:**

To identify the preparedness issues in relation to implementation of eHealth for future influenza pandemics.

**Methods:**

One hospital was selected in Australia for this study. We conducted 12 individual interviews to gather a rich data set in relation to eHealth preparedness in the context of the 2009 influenza A (H1N1) pandemic at this major teaching hospital. These participants’ views were analyzed according to five main themes: (1) challenges in present practices or circumstances for pandemic responses, which indicates a need for change, (2) healthcare providers’ exposure to eHealth, (3) organizational technological capacity to support an IT innovation for medical practices, (4) resource preparedness, and (5) socio-cultural issues in association with eHealth implementation in response to a pandemic.

**Results:**

This article reports a subset of the issues identified during the case study. These issues include, for example, poor sharing of patient health records, poor protection of patient privacy, clinicians’ concerns about IT reliability and dissatisfaction with the software in use, clinicians’ concerns about IT’s impact on professional autonomy versus having inefficient IT support, and inefficient communication across departments in the form of consultation.

**Conclusions:**

Based on discussions with the participants and interpretation of their responses, we assessed the hospital’s preparedness status and also identified areas of deficiency. Accordingly, we suggest possible solutions for the areas in need of improvement to facilitate eHealth implementation’s success. The study results will also provide policymakers at national, state and local levels with insights to refine relevant public health policies for the planning and management of pandemics from the eHealth perspective.

## Introduction

 An influenza pandemic increases morbidity and mortality across the population, threatening critical infrastructure by removing essential personnel from the workplace for weeks or months [1,2]. The 2009 influenza A (H1N1) pandemic resulted in millions of laboratory confirmed cases and over 18,000 deaths in 200 countries [3]. The pandemic strain H1N1 had similar infectivity as seasonal influenza strains in circulation in previous years. An immense burden was still placed upon health care services [4].

eHealth refers to the application of information and communication technologies (ICT) across the whole range of functions that affect health [5]. This is an emerging field at the intersection of medical informatics, public health, and business [6]. In a pandemic situation, eHealth may facilitate the pandemic response by enhancing surveillance and control activities (eg, rapid case reporting), and by facilitating the exchange of information (eg, efficient documentation and sharing of patient records) [7-10]. However, information system researchers have recognized the problems of sustainability and complexity in eHealth implementations [11,12].

The assessment of organizational preparedness for an innovation can reduce the risk of failure after introduction [13]. Preparedness in the health care context is defined as the degree to which organizations are ready for the implementation of new ICT [13,14]. If motivational forces such as health care providers’ dissatisfaction with *status quo* were not present, it would be unlikely that the innovation process would be initiated. Even though adequate motivation was present, sufficient resources would be required to allow and support steps for change. Furthermore, organizational preparedness for change is the strongest predictor of employee commitment to the organization [15]. If staff members do not possess attributes necessary for change (eg, adaptability and growth-orientation) or resist change, the change process is less likely to proceed [16]. A lack of information about health care organization preparedness for new ICT increases uncertainty for decision makers, decreases their ability to make effective decisions that would mitigate ICT innovation risks, and increases the risk of failure at critical times during a pandemic [17].

The Australian Center for Health Research Limited recognized the influenza pandemic as a threat to the hospital system, but there was no data internationally to inform the business continuity and resilience of the hospital sector. With demands for research in this area, a new collaborative project titled *Pandemic influenza, human resources and critical infrastructure dependencies: mitigating the impact on hospitals* was launched in 2009. This project brought together risk analysis, business continuity planning, and complex systems modeling methodologies based on eHealth to predict and mitigate the impact of a pandemic on the function of hospitals. As part of the project outputs, this article reports results from a case study at a major teaching hospital in New South Wales (NSW) Australia, which aimed to assess the organizational preparedness status regarding the implementation of eHealth for future influenza pandemics. The name of the hospital is not mentioned to maintain confidentiality.

## Methods

As a research strategy, case studies are used in many situations to contribute to our knowledge of individual, group, organizational, social, political, and related phenomena-it allows investigators to retain the holistic and meaningful characteristics of real-life events [18]. Case studies have a distinctive place in evaluation research [19-22]. One application is to illustrate certain topics within an evaluation in a descriptive manner [18]. In this study, one case was deliberately selected to evaluate eHealth preparedness, following a single-case design [18].

A qualitative research approach was utilized to provide a rich data set in relation to eHealth preparedness assessment, drawing on practical experiences of individuals involved in the 2009 influenza A (H1N1) pandemic response. The Medical and Community Health Research Ethics Advisory Panel, University of New South Wales approved the study protocol (Reference Number: 2011-7-10).

### Interview Guide

An interview guide was developed following a review of the literature and incorporated aspects of an integrated eHealth preparedness framework [23]. The guide provided an initial point of departure for the interview-based data collection process and examined the following areas: (1) motivational forces for change that reflect the problems identified by the evaluator and health care providers’ dissatisfaction with present practices or circumstances for pandemic responses-pandemic responses require surveillance, control, and performance of medical practices, (2) health care providers’ exposure to potential eHealth applications (engagement preparedness), including their perceived benefits and uncertainties of eHealth for a pandemic response, (3) technological preparedness as a reflection of the capacity to support an ICT innovation, (4) resource preparedness including decision makers’ knowledge of ICT implementation, supportive policies, and sufficient funding, and (5) societal preparedness in association with eHealth implementation in response to a pandemic. Communication links and partnerships need to be available within and across the organization. Questions from the interview guide (Multimedia Appendix 1) were generated to evaluate preparedness measures at the bottom level of the 5-dimension hierarchical framework, previously described by Li et al [23]. To examine the motivational forces for change, we asked the question, “were there any problems with the performance of medical practices during the influenza A H1N1 pandemic?”

### Site Selection Criteria

**Figure 1:**
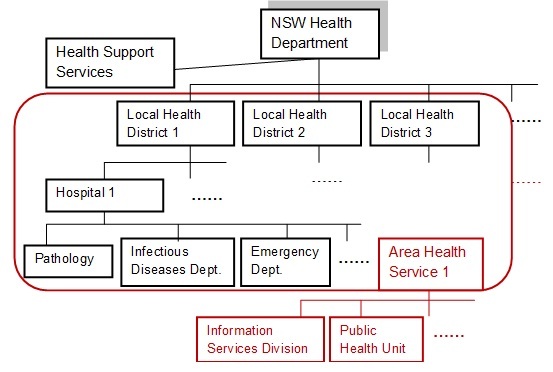
NSW Ministry of Health structure in 2011.

A number of reports and publications from the New South Wales (NSW) Ministry of Health were reviewed to understand the state health system to ensure that a representative site was selected for this case study (Figure 1). The site selected for this study followed these criteria: (1) the hospital is public and affiliated to the NSW Ministry of Health, (2) the hospital must have a large number of admitted acute patients and patients treated in the emergency department each year, (3) the hospital must have been involved in the 2009 influenza A H1N1 pandemic response, and (4) the hospital must be planning to implement a new or upgrade an existing eHealth system.

Purposive sampling was employed since the interviews required participants’ knowledge of *status quo* at the hospital to reveal its motivational, engagement, technological, resource, and societal preparedness for the prospective eHealth system implementation. Due to the nature of the data collected, three groups of participants were involved: (1) clinicians who had experience in diagnosing and reporting cases of influenza A H1N1 and who would be an end-user of the eHealth system, (2) an information technologies (IT) manager who provided IT support services during the H1N1 pandemic and who was familiar with the ICT infrastructure at the hospital (eg, the information systems in use), and (3) the chief information officer (CIO)/person who was in charge of the planning and implementation/upgrade of the eHealth system. We have also set the inclusion criteria that participants must have worked at the hospital for a minimum of 1 year, and were full-time or part-time staff (contract workers were not included).

Three interviews were piloted with a representative from each participant group of interest. The purpose was to evaluate the interview guide, for its readability, relevance, and difficulty of interpreting and answering the questions asked. The guide was modified accordingly.

### Data Collection

A hospital located in Sydney, NSW was selected for the case study. It is a major teaching hospital with almost 3000 staff and has 440 beds–an average occupancy rate (number of beds occupied) of well over 90%, ensuring that the hospital has a relatively small but highly complex caseload. Each year more than 30,000 acute patients are admitted and about 40,000 patients are treated in the emergency department. The hospital also attends to around 900,000 non-admitted patient occasions of service each year through community health, outpatients, and rural outreach services.

Potential participants from the hosptial site were nominated and contacted by email. The information services division (ISD) was a seperate entity which provided the hospital with IT-related services. Through snowball sampling, 12 eligible participants were gradually recruited from April to August 2011. The recruitment process ended once enough detailed insights were provided to reach a point of saturation with respect to the preparedness areas. Of the 12, 10 clinicians (6 medical doctors, 3 nurse managers, and 1 microbiologist) were from the infectious diseases department, emergency department, hospital epidemiology center, department of microbiology at the hospital, while the remaining 2 (the chief information officer and an IT manager) were affiliated to the ISD.

### Analysis

With the participants’ agreement and consent, all the semi-structured interviews were conducted by 1 of the investigators and recorded with a digital voice recorder. After the interviews were transcribed verbatim, the analytic process was conducted manually. A quarter of the transcripts were randomly selected and coded independently by two investigators. Subsequent discussions between them developed a list of themes. An agreed framework was then applied by 1 of the 2 investigators to code the remainder of the transcripts and the themes were further modified. Based on the themes finally identified, all of the transcripts were analyzed. The analysis results were then discussed with the other authors. Lastly, modifications were made according to those comments and feedback.

## Results

The NSW Ministry of Health divided the state into eight separate regions, which were called Area Health Services (AHS). Across the AHS where the hospital was located, there were more than 300 information systems in use for various purposes including care delivery, logistics, and finance. One widely used system within the AHS was the electronic medical records (EMR). The plan for the EMR was commenced in February 2007 and the Cerner EMR suite was selected. The key features of the system included scheduling of patients for operating theatres and retrieval of radiology and pathology results. At the hospital selected for the case study, EMR implementation occurred from October 2009 to June 2011. Until July 2011, the EMR had been utilized in 955 clinics across 16 hospitals for clinical practices. According to respondents from the ISD, there was still the challenge of sharing patient health information across hospitals even at the state level due to the absence of a unique patient identifier.

You may go to two different hospitals, having different identifiers, so EMRs may be created for you in the Hospital A; separately you may go into Hospital B when you’ve gone back home and Hospital B may say we don’t have access to that information at this point in time … you’ve got all this information being collected in different hospitals. What you need is a unique identifier, which sits at the state level. Once that becomes available, it becomes easier.

Respondents from the ISD indicated that IT development had never stopped and the ISD was continuously looking for opportunities to facilitate better patient care outcomes. They pointed out that it was planned to implement a new IT system, which was referred to as the Antimicrobial Stewardship System (ASS). The project team had involved health professionals from the infectious disease department of a few hospitals, as well as pharmacists. According to the interviewed clinicians, the system would monitor antibiotic use, cut down patient care cost by reducing inappropriate antibiotic prescriptions, and also improve patient treatment outcomes.

One clinician indicated that there was a list of restricted antibiotics which clinicians had to apply to use. The system would provide clinicians with an automatic authorization to prescribe these antibiotics once they gave the right indication. There would be a way of tracking that information if somebody tried to “game” the system. A few interviewed clinicians pointed out that inappropriate antibiotic prescriptions could occur, for example, by unnecessary use of antibiotics for a patient’s condition or expensive antibiotics, and prescribing a larger amount, for a longer duration or a wider spectrum of antibiotic use than was required. A couple of clinicians perceived and suggested that the ASS could improve antimicrobial prescriptions and specificity in a timely fashion for only the duration that was required.

The following sections report on a subset of the case study’s results, which identified issues related to the hospital’s preparedness for the implementation of eHealth as well as response to influenza pandemics.

### Motivational Preparedness

#### Capturing Alerts Issued by Public Health Units

Health alerts during the 2009 pandemic were issued by both the state Department of Health and the Local Health District. Clinicians received these alerts through emails, facsimile, the hospital intranet website, and the state bulletins. The alerts contained information on H1N1 updates (eg, the number of cases, updated case definitions, current and accepted best practice). Clinicians pointed out that there was a lack of reliable information at the beginning of the pandemic, followed by an unmanageable number of emails with excessive, unfiltered, and repetitive information on H1N1 at the later stages of the pandemic. Clinicians suggested that summarized updated information placed at workstations would have been more effective than multiple alerts sent on the same topic to each clinician.

#### Documentation Efficiency

When commenting on the statement that the retrieval, update, and storage of patient health records were inefficient during the 2009 pandemic, the majority of the interviewed clinicians agreed at varying degrees. Some clinicians highlighted that EMR were implemented at the hospital towards the end of the pandemic in 2009, and that a lot of information (eg, previous clinical assessments, medication history, and previous prescriptions) required for clinical practices was not available in the EMR. Consequently, clinicians still had to retrieve information from the understaffed medical records department where paper medical records were stored. This was a time consuming process and not ideal especially during a pandemic.

#### Completeness and Accuracy of Patient Health Records

A couple of interviewed clinicians deemed that complete and accurate patient medical history was not important for clinical decision making in the circumstances of the 2009 pandemic. During the pandemic, the majority of the patients affected were younger and therefore had few medical complications. As such, a complete history of the patient was often unnecessary, and that the appropriate medical treatment could be given based on their clinical presentations at the time. One of the others argued against that, indicating that information on “whether the patient was pregnant or got diabetes or other kinds of diseases” was important for clinical decision making in the flu setting.

Most clinicians reported inaccurate and incomplete patient medical records even with EMR in use. Some explained that the patient’s medical history was generated by various health professionals at multiple locations such as hospitals and GPs and the accuracy and completeness was dependent on “how well the clinical record was made originally” and on how all that information could be shared from different locations. They needed to have “a universal aligned EMR travel between hospital and GPs”. When asked what information was available in the EMR at the hospital, the interviewed clinicians mentioned emergency assessment documentation, operating theatre reports, pathology and radiology results, and some discharge information, but pointed out that medical officers’ assessments (eg, past diagnoses, medication and other clinical notes) were excluded.

#### Patient Privacy and Information Security

Access to eHealth applications such as Laboratory Information System (LIS) and EMR, required a username and password. Nevertheless, clinicians across departments could look up and access anybody’s medical record at any time, leaving patient information uncontrolled. Common access to patient records between clinicians with different designations could breach the patients’ confidentiality. One clinician argued against that electronic trails of system access and data operations that had been in use – what a user retrieved from the systems was recorded with time stamps within the workstation or in a repository, and assessment could then be subsequently conducted on those records. A senior clinician from the infectious disease department suggested that “the rules around confidentiality” should be better specified and that the EMR usage should conform to those rules.

#### Correctness of Diagnoses

Although the majority thought that diagnosis could have been incorrectly given due to lack of patients’ full history, a small number of the interviewed clinicians disagreed. They argued that the clinical diagnosis could provisionally be made even before confirmation from the laboratory test, if patients presented the signs and clinical symptoms of the H1N1 case definition (eg, high fever, cough, and sore throat).

Various reasons were given for what could cause incorrect diagnoses, including poor clinical history taken, language barriers resulting in misinformation between patients and doctors for clinical decision making, mislabelled samples, delays on processing and testing of lab specimens, false positive or false negative laboratory test results, data entry errors into the LIS, and inexperienced practice or irresponsible clinicians.

People were not diagnosed with the pandemic flu who probably had the flu, but on the basis of negative lab test, were not considered to have the disease. That was poor understanding of the performance of the test result.

If a rapid test comes back negative, some staff initially would go ‘no, they don’t need isolation’. They obviously had an influenza-like illness, people go ‘but that test is negative’, don’t take into consideration what’s happening.

#### Appropriateness of Prescriptions

Few clinicians interviewed believed that no errors in prescriptions took place at the hospital. They explained that access to Tamiflu for influenza A (H1N1) had to be approved by the infectious disease department and also that a very standard treatment dose was specified in the case definition. Others argued that “there is always an error margin” around prescriptions.

A number of reasons could have led to errors. During times of a pandemic, clinicians were often bombarded with an overwhelming amount of questions simultaneously, which could increase work pressure and distractions, leading to forgotten medication orders or transcribing errors. There could also be issues with inadequate knowledge of medication, which could result in prescribing the wrong drugs for the wrong diagnosis, incorrect doses, and inappropriately assigned treatment duration. Due to the high level of pressure during these times, clinicians can prescribe a drug without careful consideration of contraindications (eg, patient’s allergy history). Finally, there could also be dispensing errors at the pharmacy due to overwhelming volumes of prescriptions, illegible prescriptions, and a lack of time and resources to check for these errors, which they would normally have done.

### Engagement Preparedness

#### Clinicians’ Concern About Reliability of IT

The majority of the interviewed clinicians disagreed with the statement that information technology is always reliable, indicating that technology glitches and downtime had resulted in interruption and inconvenience in their clinical practice. Paper trail records were supported in addition to IT measures in case of failure of the technology in use.

Apart from IT itself, information available through the technology could also be unreliable, as some clinicians added. They explained that the reliability was dependent on the information source, which could be outdated, or there could be typing errors.

#### Clinicians’ Concern About IT’s Impact on Professional Autonomy

The majority disagreed with the statement that their professional autonomy in health care systems was not their concern after the ASS was introduced to health care practice. One explained that the EMR provided a drop-down list of diagnoses when patients were discharged. If the diagnosis was not in the list, clinicians were forced to select an incorrect choice from the list instead of being allowed to type in the actual diagnosis. These imposed operations caused the loss of their professional autonomy. A couple of clinicians added that, with pervasive information technology at the health care facility, clinicians’ practice became dependent on IT professionals’ support and frequent interactions occurred between these professional groups. “That’s culture change”, commented by one of the others who agreed with the statement. When referring to the ASS, 1 clinician from the emergency department argued that medical doctors needed to have the freedom to prescribe what they thought was necessary in the event of an emergency–“anything that will restrict us would be opposed quite strongly”. A few argued further that with a guideline or protocol, the proven standard indication might not be appropriate for a patient’s specific condition.

Some others agreed that the implementation of the ASS could challenge medical doctors’ autonomy. However, they argued that better patient care outcomes and patient safety should be the primary concern of professional autonomy without required relevant clinical knowledge. .

### Technological Preparedness

#### Clinicians’ Dissatisfaction with the Software in Use

According to the IT manager, about 190 IT applications were in use within the AHS. At the hospital, available clinical and non-clinical applications included, the EMR, LIS, picture archiving and communication system, community health information management system for health workers visiting patients at home, human resource management system, car booking system, Oracle financials (solutions to a wide range of long- and short-term accounting system issues), and payroll management system.

Most interviewed clinicians were dissatisfied with the hospital IT systems and pointed out problems such as: (1) integration issues–although pathology and radiology results had been integrated with the EMR, external paper medical histories still existed and had not been integrated, therefore, clinicians had to look at the EMR and also check paper records (eg, “drug orders in the clinical note”) for clinical decision making, (2) poor response time–wait times during loading of EMR, log in, redundant pop-up questions confirming identity of user, accessing internal links, log out, and shut down of the system, (3) unfriendly user interface–many felt that the interface of the EMR system was not intuitive, and (4) inconvenient secondary use of available clinical data–although clinicians could efficiently share laboratory test results on a single patient basis through the LIS, it was difficult to extract and collect these data on a population basis to do overall audits from the infectious diseases’ perspective as the LIS did not have that capability.

A few clinicians indicated that due to some of these limitations of the IT systems, the clinician-patient relationship could be interfered. Clinicians now had to spend more time on the computer to handle these systems rather than in face-to-face contact and conversation with the patient.

You walked in the department before, you would see a few people at the computers, a lot of people with patients. Now, it’s the other way around: a few people with patients, a lot of people at the computers … imagine a patient is screaming in pain and wants your attention.

#### Inefficient IT Support Perceived by Clinicians

The procedure in the event of an IT systems failure is to contact the Statewide Service Desk (SWSD) and log a call (ie, ticket). The SWSD is a centralised service desk for health facilities across NSW and is run by the Health Support Services. Based on the information provided by the caller at the first point of contact, the SWSD operator makes a brief analysis and electronically allocates a ticket through the SWSD system to 1 of 6 groups at the ISD: (1) project planning group who provide IT project consultation services and manage procurement, (2) communication group who ensure that the computer network is working, (3) application support group who look after the applications provided by the AHS, specifically dealing with interface problems between EMRs and the patient administration system, (4) desktop support group who installs the required software in person, (5) technical services group who takes care of hardware and the data center such as data backup and email accounts, and (6) client support service group who provide IT support such as troubleshooting to the users.

Subsequently, the group, on behalf of the health facility to which the caller is affiliated, manages the ticket. The IT manager indicated that the client support service group (27 people) alone managed over 2,000 calls a month. He commented that the ticket allocation could sometimes be inappropriate due to the misdiagnosis of problems by the operator. He explained that email access failure, for example, could be caused by a faulty network card or a dysfunctional port; the former should be taken care of by the technical services group whereas the latter should be by the communication group. If the operator did not ask the caller appropriate questions, the ticket could be allocated to the wrong group and consequently it would take longer than it should to solve the problem.

When asked whether IT support for troubleshooting was efficient, half of the interviewed clinicians agreed (“absolutely fantastic”) while the other half gave completely opposite views (“absolutely pathetic, terrible”). Most the clinicians who disagreed argued that there was inefficient communication with the SWSD. A few explained that it took minutes before they could even talk to someone and had to enter a lot of information before they could proceed. Some further explained that the line could be busy and they needed to log a call, waiting for SWSD operators to ring them back. If they missed the call back from the SWSD, they had to call again and re-log the call. Once the call was allocated to the right person, the problem could be solved efficiently by either remote or on-site support.

### Societal Preparedness

The CIO indicated that there was always cooperation between departments of interest. The ISD had been working closely with clinicians from different departments and involving them through the implementation process. When asked to comment on the statement that communication across the department was efficient in the form of consultation, the minority of clinicians indicated their agreement. Some argued that there could be delays in the consultation process, for example, due to delays in sending out requests–clinicians with insufficient medical specialty knowledge might not realize the need for a consultation with specialist staff early on, and as a result the request would be made later than it should. Delays in response to requests are also possible; if the request was sent through the paging system, for example, and the recipient’s page was inaccessible at that point of time, a delayed response would occur.

Some pointed out that communication efficiency was also dependent on the professional relationship of clinicians across departments who are involved in the consultation and involvement of key people who have a cross-department role for cooperation.

When you need to actually draw departments together (eg, consultation), it’s better to speak to key people. You might send out a group email; it’s important that you have the right person signing it; otherwise it will take no notice. Depends on the author (smile). So that’s really important.

## Discussion

Based on discussions with the participants and interpretation of their responses, we have identified the areas of deficiency in the hospital’s preparedness for the implementation of eHealth (eg, the ASS) and response to future influenza pandemics. Table 1 summarizes these areas of deficiency with possible solutions.

**Table 1:** Identified areas of deficiency and suggestions.

Areas of deficiency	Suggestions
Timeliness of issuing and capturing health alerts	(a) Electronic case reporting rather than by telephone or in writing.(b) Exploring the modality of alerts being issued (eg, a hotlink on the desktop of clinicians’ workstations).
Documentation efficiency	Scanning, indexing, and integrating external paper-based documents into the EMR.
Sharing of patient health records and protection of patient privacy	(a) Applying a unique patient identifier to facilitate collaborative health care delivery across facilities.(b) Defining what information needed to be shared with whom and in which way.(c) Adopting the role-based access control (RBAC).
Correctness of diagnoses	(a) In the circumstances of a pandemic: providing clinicians with updated case definitions with check-box criteria.(b) For medical practice in general: using a set of logical if-then rules extracted from medical guidelines.
Appropriateness of prescriptions	(a) Using the ASS being implemented.(b) Exploring other options such as automatic check for contraindication with complete and updated patient information.
Clinicians’ concerns about IT reliability and dissatisfaction with the software in use	Requiring a more strategic methodology for its design and service management, such as an Eight-Stage Service Design and Management Model and House of Quality Matrix.
Clinicians’ concerns about IT’s impact on autonomy versus having inefficient IT support	(a) Requiring more operators the SWSD for a particular time period (eg, at the early implementation phase of a new system).(b) Providing SWSD operators with more IT-related training and education to correctly diagnose and allocate technical problems to ISD groups.
Inefficient communication across departments in the form of consultation	Involving key people for cross-department cooperation.

Regarding motivational preparedness, identification of the challenges within present practices for pandemic responses indicates a need for change. Perceived needs by health care providers impact on their behavioral intention to adopt and use an eHealth system [24,25]. In response to the issues raised during the interviews, some of the broad requirements for IT development in order to improve response to a future pandemic are outlined below.

### Timeliness of Capturing Alerts Issued by Public Health Units

To issue reliable health alerts in a timely fashion, public health units initially needed to collect case information from reporting sources (eg, clinicians). If alerts were issued by email, clinicians would not be able to access them in real time due to overloaded clinical practices on the floor. To improve the timeliness of capturing alerts, case reporting should be made electronic rather than by telephone or in writing–case notification could be made immediately after clinicians’ diagnosis. Furthermore, there is a need to explore the modality of alerts being issued (eg, a hotlink on the desktop of clinicians’ workstations or SMS messages, as suggested by the interviewed clinicians).

### Documentation Efficiency

Although EMRs were available, external paper-based documents (eg, patient medical history stored at the medical records department before the EMR implementation) were still required for current clinical decision making. Clinicians pointed out that retrieval of these documents was inefficient. The paper documents should be scanned, indexed, and integrated into the EMR.

### Sharing of Patient Health Records and Protection of Patient Privacy

The interviewed clinicians indicated that it was difficult to share patient health records particularly across the area health services and between states. A unique patient identifier (ie, National Health Identifier, NHI) should be applied to facilitate collaborative health care delivery within and across service settings in the country. A variety of clinicians from multiple service settings should be assured of access to patient medical history when required, but with the RBAC utilized to protect patient privacy and information security. Further exploration was required to define what information needed to be shared with whom and in which way.

### Correctness of Diagnoses

The interviewed clinicians explained the reasons why incorrect diagnoses happened and named a few (eg, false positive and false negative laboratory test results, inexperienced practice or irresponsible clinicians). Clinicians suggested that the ICT application could reduce diagnostic errors by providing updated case definitions to clinicians with check-box criteria in the circumstances of a pandemic. For the medical practice in general, ICT options should be explored, for example, using a set of logical if-then rules extracted from medical guidelines.

### Appropriateness of Prescriptions

The reasons varied for prescription errors, as clinicians explained (eg, inadequate knowledge of medication and absence of the consideration of contraindications). The ASS is an example of how ICT can be applied to reduce prescription errors, but it will only be used for antibiotics. Options to decrease prescription errors need to be further explored (eg, automatic check for contraindication with complete and updated patient information).

### Other Areas of Deficiency

Deficiencies in the hospital’s preparedness were also identified under other three main themes and needs to be addressed. Many clinicians correlated their doubt about IT reliability with their frustration from or dissatisfaction with the IT systems in use (eg, poor response time and unfriendly user interface). Negative IT experience can cause them technology phobia and thus inhibit their adoption intention of a new eHealth system [26,27]. Any IT system in the future will require a more strategic methodology for its design and service management, such as an Eight-Stage Service Design and Management Model [28] and House of Quality Matrix [29].

Some clinicians perceived and indicated that due to the increasing penetration of information technology into health care settings, clinical practices have become more and more dependent on IT support. Nevertheless, the support was perceived as inefficient as a result of inefficient communication with the SWSD. Efficient technical support particularly for troubleshooting takes a predominant role in smoothing clinicians’ re-engineered job routine and overcoming their technology phobia, and consequently can facilitate their acceptance and use of a new eHealth system [30,31]. Clinicians indicated that if there was no operator available, which was often the case, they had to be on hold for variable lengths of time to report their problem. As a possible solution, more operators should be put on duty at a particular time period at the early implementation phase of a new system. The IT manager also pointed out that due to insufficient IT knowledge among some SWSD operators, misdiagnosis of problems took place, and consequently problems were misallocated to ISD groups. He remarked that the misallocation resulted in a decrease of IT support efficiency. To address this issue, more IT-related training and education should be provided to these operators.

Clinicians reported that there were delays both in sending out consultation requests due to senders’ insufficient medical specialty knowledge and in responding to requests due to some other facts in relation to the recipients (eg, performing an operation). In the context of a pandemic response or eHealth implementation, cooperation, and communication is often required between medical departments and the IT team to share ideas, address concerns, alleviate fears and mediate tensions amongst involved clinicians and IT staff [32]. A senior nurse from the hospital epidemiology center suggested that it was necessary to involve key people at least for cross-department cooperation, which could facilitate two-way communication.

### Limitations

This article examined organizational and health care providers’ preparedness at a hospital in NSW for the implementation of the ASS in the context of the 2009 pandemic. The results of this case study may be limited due to participants’ over-reporting or their recall bias. The three groups of participants may have over-reported their preparedness in order to avoid embarrassment or judgement. We attempted to minimize any bias in the interpretation of the interview data by having it reviewed by two investigators.

### Contributions

eHealth preparedness assessment helps the decision maker at a health care organization to be well-informed of deficient areas in preparedness, and therefore serve as a guide for preventive action to combat the failure to innovate [13,14]. A few studies [33,34] have been found in the literature on the development of a framework for eHealth preparedness assessment. These frameworks were developed from different perspectives. Most studied components reflected health care providers’ and organizational perspectives, but these components were different from one framework to another [23]. By integrating these components, a 5-dimension framework [23] provided a guideline for eHealth preparedness assessment in the context of a pandemic. This integrated framework has not yet been applied in real health care settings. Also, there is no study internationally on the evaluation of eHealth preparedness in an organizational context. Regarding theoretical contributions, our study has demonstrated the applicability of the integrated framework in a real health care setting and also provides the medical informatics audience with an example of how eHealth preparedness assessment can be conducted in an organizational context. We believe that these theoretical contributions will prompt further investigation among practitioners and academicians on organizational preparedness for the implementation of e Health systems.

In practice, our findings and discussions may assist decision makers in the organizations to take action to address deficient areas in their preparedness and, as a result, facilitate the eHealth implementation success. Pandemic preparedness planning is necessitated during the inter-pandemic period to enable countries to be prepared to recognize and manage an influenza pandemic [35]. These reported findings may also provide policymakers at national, state, and local levels with empirical evidence and insights in order to refine relevant public health policies for the planning and management of pandemics from the ICT perspective. For example, a deficient area was found in the protection of patient privacy. The national and state governments need to enact and implement policies to address this issue and clearly define what information needs to be shared with whom and in which way to control the access. The implementation of those eHealth solutions would more likely succeed if there is a RBAC control feature in compliance with these policies. Health care providers and patients’ concern over the security of patient information and protection of patient privacy has been identified in the literature as one of the most significant factors influential to their acceptance of eHealth [36].

### Future Work

In the future, similar studies can be conducted at various health care settings (eg, residential aged care centers and primary health care centers) to manage and plan the implementation of varied and specific eHealth systems such as electronic health records, elearning, chronic illness management, telecardiology, teleradiology, and teledermatology.

## Multimedia Appendix 1

Interview Guide.

## Abbreviations

AHS: area health services
ASS: antimicrobial stewardship system
EMR: electronic medical records
ICT: information and communication technologies
ISD: information services division
IT: information technologies
LIS: laboratory information system
NHI: national health identifier
NSW: New South Wales, Australia
RBAC: role-based access control
SWSD: statewide service desk
