# Supplementary material for: Issues Regarding the Implementation of eHealth: Preparing for Future Influenza Pandemics
Source: Interact J Med Res. 2012 Dec 6;1(2):e20. doi: 10.2196/ijmr.2357 (PMC3626132; doi:10.2196/ijmr.2357)
Supplement: Supplementary file 1 [file ijmr_v1i2e20_app1.pdf]

A. INTERVIEW GUIDE WITH CLINICIANS FOR CASE STUDY

CODE: DATE:

Interview Instructions

The objective of this project is to assess the E-Health preparedness for pandemic response in this hospital. This interview will take approximately 45 minutes to complete and will be recorded. All the collected data is confidential and will only be used in this research. We understand that you are very busy, so we appreciate your participation in this research.

An example of the questions:

This hospital was involved in influenza A H1N1 pandemic response. Regarding this statement, do you agree or disagree?

strongly disagree	disagree	mildly disagree	neutral	mildly agree	agree	strongly agree
☐	☐	☐	☐	☐	☐	☒

A follow up question(s) may be asked to provide clarity around the interviewee's answer. For example: "*In which way was the hospital involved?*"

Interview face sheet

Age: [________] (years)

Sex: ☐ Male ☐ Female

Number of years working in healthcare [________] (years)

What ward/department do you work in? ………………………..

Current position at hospital, specify ………………………..

Number of years in current position: [________] (years)

Interview Guide

Q1. During the influenza A H1N1 pandemic were there any problems with the hospital's participation in public health surveillance? Do you think that IT systems can facilitate public health surveillance activities?

Q1.1. Case reporting to the state or local health department was not rapid and timely.
Do you agree or disagree?

strongly disagree	disagree	mildly disagree	neutral	mildly agree	agree	strongly agree
☐	☐	☐	☐	☐	☐	☐

IT can facilitate rapid case reporting to public health units.
Do you agree or disagree?

strongly disagree	disagree	mildly disagree	neutral	mildly agree	agree	strongly agree
☐	☐	☐	☐	☐	☐	☐

Q1.2. Patient information (e.g., lab test results) was inaccessible after the case was reported.
Do you agree or disagree?

strongly disagree	disagree	mildly disagree	neutral	mildly agree	agree	strongly agree
☐	☐	☐	☐	☐	☐	☐

IT can improve the accessibility of patient information (e.g., lab test results) after the case is reported.
Do you agree or disagree?

strongly disagree	disagree	mildly disagree	neutral	mildly agree	agree	strongly agree
☐	☐	☐	☐	☐	☐	☐

Q1.3. Alerts issued by public health units were not timely captured.
Do you agree or disagree?

strongly disagree	disagree	mildly disagree	neutral	mildly agree	agree	strongly agree
☐	☐	☐	☐	☐	☐	☐

IT can assist with capturing timely alerts issued by public health units.
Do you agree or disagree?

strongly disagree	disagree	mildly disagree	neutral	mildly agree	agree	strongly agree
☐	☐	☐	☐	☐	☐	☐

Q2. Were there any problems with the performance of medical practices during the influenza A H1N1 pandemic? Do you think that IT systems can facilitate patient care practices?

Q2.1. The retrieval, update, and storage of patient health records were inefficient.
Do you agree or disagree?

strongly disagree	disagree	mildly disagree	neutral	mildly agree	agree	strongly agree
☐	☐	☐	☐	☐	☐	☐

IT can facilitate rapid documentation, i.e., retrieval, update and storage of patient health records.
Do you agree or disagree?

strongly disagree	disagree	mildly disagree	neutral	mildly agree	agree	strongly agree
☐	☐	☐	☐	☐	☐	☐

Q2.2. Complete and accurate patient records were unavailable for clinical decision making.
Do you agree or disagree?

strongly disagree	disagree	mildly disagree	neutral	mildly agree	agree	strongly agree
☐	☐	☐	☐	☐	☐	☐

IT can facilitate sharing of required patient information for patient care practice.
Do you agree or disagree?

strongly disagree	disagree	mildly disagree	neutral	mildly agree	agree	strongly agree
☐	☐	☐	☐	☐	☐	☐

Q2.3. Access to patient information by various healthcare providers was uncontrolled.
Do you agree or disagree?

strongly disagree	disagree	mildly disagree	neutral	mildly agree	agree	strongly agree
☐	☐	☐	☐	☐	☐	☐

IT can control the access to patient records.
Do you agree or disagree?

strongly disagree	disagree	mildly disagree	neutral	mildly agree	agree	strongly agree
☐	☐	☐	☐	☐	☐	☐

Q2.4. Incorrect diagnoses happened in the hospital.
Do you agree or disagree?

strongly disagree	disagree	mildly disagree	neutral	mildly agree	agree	strongly agree
☐	☐	☐	☐	☐	☐	☐

IT can reduce clinical diagnostic errors.
Do you agree or disagree?

strongly disagree	disagree	mildly disagree	neutral	mildly agree	agree	strongly agree
☐	☐	☐	☐	☐	☐	☐

Q2.5. Errors in prescriptions took place in the hospital.
Do you agree or disagree?

strongly disagree	disagree	mildly disagree	neutral	mildly agree	agree	strongly agree
☐	☐	☐	☐	☐	☐	☐

IT can reduce prescription errors (e.g., by automatic check of drugs interaction and contraindication).
Do you agree or disagree?

strongly disagree	disagree	mildly disagree	neutral	mildly agree	agree	strongly agree
☐	☐	☐	☐	☐	☐	☐

Q2.6. Software tools were unavailable to assist with answering patients' questions.
Do you agree or disagree?

strongly disagree	disagree	mildly disagree	neutral	mildly agree	agree	strongly agree
☐	☐	☐	☐	☐	☐	☐

IT can assist with answering patients' questions (e.g., by a customised search engine).
Do you agree or disagree?

strongly disagree	disagree	mildly disagree	neutral	mildly agree	agree	strongly agree
☐	☐	☐	☐	☐	☐	☐

Q3. Planning is currently underway at this hospital to implement a new IT system. Do you know what this system is going to be used for?

Do you agree or disagree with the following statements regarding using these systems?
Q3.1. Information technology is always reliable.

strongly disagree	disagree	mildly disagree	neutral	mildly agree	agree	strongly agree
☐	☐	☐	☐	☐	☐	☐

Q3.2. Are you confident that you've got sufficient IT skills to be able to operate the new system.

strongly disagree	disagree	mildly disagree	neutral	mildly agree	agree	strongly agree
☐	☐	☐	☐	☐	☐	☐

Q3.3. High investment and/or low reimbursement are not your concern with using the new system.

strongly disagree	disagree	mildly disagree	neutral	mildly agree	agree	strongly agree
☐	☐	☐	☐	☐	☐	☐

Q3.4. The new system can be aligned with present clinical practice processes and activities.

strongly disagree	disagree	mildly disagree	neutral	mildly agree	agree	strongly agree
☐	☐	☐	☐	☐	☐	☐

Q3.5. Professional livelihood or autonomy to healthcare systems or both is not your concern after the new system is introduced to healthcare practice.

strongly disagree	disagree	mildly disagree	neutral	mildly agree	agree	strongly agree
☐	☐	☐	☐	☐	☐	☐

Q3.6. You are willing to make initial investment of extra time in the training for using this new system.

strongly disagree	disagree	mildly disagree	neutral	mildly agree	agree	strongly agree
☐	☐	☐	☐	☐	☐	☐

Q4. What do you think of the current IT infrastructure in the hospital?

Q4.1. IT equipment (e.g., computer terminals and photocopiers) is located where it is convenient for you to use.
Do you agree or disagree?

strongly disagree	disagree	mildly disagree	neutral	mildly agree	agree	strongly agree
☐	☐	☐	☐	☐	☐	☐

Q4.2. You are satisfied with the software in use in the hospital.
Do you agree or disagree?

strongly disagree	disagree	mildly disagree	neutral	mildly agree	agree	strongly agree
☐	☐	☐	☐	☐	☐	☐

Q4.3. The Internet access is reliable and stable.
Do you agree or disagree?

strongly disagree	disagree	mildly disagree	neutral	mildly agree	agree	strongly agree
☐	☐	☐	☐	☐	☐	☐

Q4.4. IT support for troubleshooting is efficient.
Do you agree or disagree?

strongly disagree	disagree	mildly disagree	neutral	mildly agree	agree	strongly agree
☐	☐	☐	☐	☐	☐	☐

Q4.5. IT support for troubleshooting is effective.
Do you agree or disagree?

strongly disagree	disagree	mildly disagree	neutral	mildly agree	agree	strongly agree
☐	☐	☐	☐	☐	☐	☐

Q5. What's your IT background?

Q5.1. You use computers frequently.
Do you agree or disagree?

strongly disagree	disagree	mildly disagree	neutral	mildly agree	agree	strongly agree
☐	☐	☐	☐	☐	☐	☐

Q5.2. You use the Internet frequently.
Do you agree or disagree?

strongly disagree	disagree	mildly disagree	neutral	mildly agree	agree	strongly agree
☐	☐	☐	☐	☐	☐	☐

Q5.3. You use emails frequently for social networks and communication purpose.
Do you agree or disagree?

strongly disagree	disagree	mildly disagree	neutral	mildly agree	agree	strongly agree
☐	☐	☐	☐	☐	☐	☐

Q5.4. You use other devices available at work (e.g., photocopiers) frequently.
Do you agree or disagree?

strongly disagree	disagree	mildly disagree	neutral	mildly agree	agree	strongly agree
☐	☐	☐	☐	☐	☐	☐

Q5.5. You have had training or direct experience in using IT systems for patient care or public health surveillance.
Do you agree or disagree?

strongly disagree	disagree	mildly disagree	neutral	mildly agree	agree	strongly agree
☐	☐	☐	☐	☐	☐	☐

Q6. Is communication in the hospital efficient?

Q6.1. In your department, communication amongst healthcare providers is efficient for sharing locally relevant content and delivering care services.
Do you agree or disagree?

strongly disagree	disagree	mildly disagree	neutral	mildly agree	agree	strongly agree
☐	☐	☐	☐	☐	☐	☐

Q6.2. Communication across departments is efficient in the form of consultation.
Do you agree or disagree?

strongly disagree	disagree	mildly disagree	neutral	mildly agree	agree	strongly agree
☐	☐	☐	☐	☐	☐	☐

B. INTERVIEW GUIDE WITH IT OFFICERS FOR CASE STUDY

CODE: **DATE:**

Interview Instructions

The objective of this project is to assess the E-Health preparedness for pandemic response in this hospital. This interview will take approximately 30 minutes to complete and will be recorded. All the collected data is confidential and will only be used in this research. We understand that you are very busy, so we appreciate your participation in this research.

An example of the questions:

This hospital was involved in influenza A H1N1 pandemic response. Regarding this statement, do you agree or disagree?

strongly disagree	disagree	mildly disagree	neutral	mildly agree	agree	strongly agree
☐	☐	☐	☐	☐	☐	☒

A follow up question(s) may be asked to provide clarity around the interviewee's answer. For example: "*In which way was the hospital involved?*"

Interview face sheet

Age: [________] (years)

Sex: ☐ Male ☐ Female

Number of years working in healthcare [________] (years)

What ward/department do you work in? ………………………..

Current position at hospital, specify ………………………..

Number of years in current position: [________] (years)

Interview Guide

Q1. It was advised to us, that this hospital is going to implement a new E-Health system. What is this system going to be used for?

Q2. Is existing hardware able to support the new E-Health implementation?

Q2.1. Which type of hardware is required to implement the new E-Health system?

Q2.2. The hardware required for the new E-Health implementation is available.
Do you agree or disagree?

strongly disagree	disagree	mildly disagree	neutral	mildly agree	agree	strongly agree
☐	☐	☐	☐	☐	☐	☐

Q2.3. Existing hardware in the hospital functions reliably.
Do you agree or disagree?

strongly disagree	disagree	mildly disagree	neutral	mildly agree	agree	strongly agree
☐	☐	☐	☐	☐	☐	☐

Q3. Is existing software able to support the new E-Health implementation?

Q3.1. Clinical/non-clinical software is available (e.g., applications to send emails).
Do you agree or disagree?

strongly disagree	disagree	mildly disagree	neutral	mildly agree	agree	strongly agree
☐	☐	☐	☐	☐	☐	☐

Q3.2. What are the information systems/software packages in use by doctors in the hospital?

Q4. Is existing network able to support the new E-Health implementation?

Q4.1. How fast is the Internet connection? (e.g., Kilobits per second [Kbps=10^3], Megabits per second [Mbps=10^4], Gigabits per second [Gbps=10^6], Megabytes per second [MBps; 1 byte=8 bits])

Q4.2. High bandwidth access is available for the new E-Health system.
Do you agree or disagree?

strongly disagree	disagree	mildly disagree	neutral	mildly agree	agree	strongly agree
☐	☐	☐	☐	☐	☐	☐

Q5. Are existing IT personnel able to support the new E-Health implementation?

Q5.1. What are the IT personnel’s responsibilities in the hospital?

Q5.2. IT support for troubleshooting is available.
Do you agree or disagree?

strongly disagree	disagree	mildly disagree	neutral	mildly agree	agree	strongly agree
☐	☐	☐	☐	☐	☐	☐

What’s your IT troubleshooting experience in the influenza A H1N1 pandemic?

C. INTERVIEW GUIDE WITH THE CIO FOR CASE STUDY

CODE: **DATE:**

<u>Interview Instructions</u>

The objective of this project is to assess the E-Health preparedness for pandemic response in this hospital. This interview will take approximately 40 minutes to complete and will be recorded. All the collected data is confidential and will only be used in this research. We understand that you are very busy, so we appreciate your participation in this research.

An example of the questions:

This hospital was involved in influenza A H1N1 pandemic response. Regarding this statement, do you agree or disagree?

strongly disagree	disagree	mildly disagree	neutral	mildly agree	agree	strongly agree
☐	☐	☐	☐	☐	☐	☒

A follow up question(s) may be asked to provide clarity around the interviewee's answer. For example: "*In which way was the hospital involved?*"

Interview face sheet

Age: [________] (years)

Sex: ☐ Male ☐ Female

Number of years working in healthcare [________] (years)

What ward/department do you work in? ………………………..

Current position at hospital, specify ………………………..

Number of years in current position: [________] (years)

Interview Guide

Q1. It was advised to us, that this hospital is going to implement a new E-Health system. What is this system going to be used for?

Q2. Have you got specific knowledge about implementing an E-Health system?

Q2.1 You're knowledgeable in the management of IT project procurement (e.g., outsourcing).
Do you agree or disagree?

strongly disagree	disagree	mildly disagree	neutral	mildly agree	agree	strongly agree
☐	☐	☐	☐	☐	☐	☐

Q2.2. You're knowledgeable in continuing professional development to healthcare providers (e.g., established mechanism of knowledge transferring between staff – training).
Do you agree or disagree?

strongly disagree	disagree	mildly disagree	neutral	mildly agree	agree	strongly agree
☐	☐	☐	☐	☐	☐	☐

Q3. Are there supportive policies in place for the new E-Health systems implementation?

Q3.1. Policies for healthcare providers' reimbursement are available.
Do you agree or disagree?

strongly disagree	disagree	mildly disagree	neutral	mildly agree	agree	strongly agree
☐	☐	☐	☐	☐	☐	☐

Q3.2. Policies for healthcare providers' liability are available.
Do you agree or disagree?

strongly disagree	disagree	mildly disagree	neutral	mildly agree	agree	strongly agree
☐	☐	☐	☐	☐	☐	☐

Q3.3. Policies for jurisdiction and privacy are available.
Do you agree or disagree?

strongly disagree	disagree	mildly disagree	neutral	mildly agree	agree	strongly agree
☐	☐	☐	☐	☐	☐	☐

Q4. Is there sufficient funding for both initial implementation processes and ongoing maintenance of the new E-Health system? Who will provide the funding: government or other stakeholders?

Q4.1. There is sufficient funding for purchase/upgrade of ICT infrastructure.
Do you agree or disagree?

strongly disagree	disagree	mildly disagree	neutral	mildly agree	agree	strongly agree
☐	☐	☐	☐	☐	☐	☐

Q4.2. There is sufficient funding for project management (e.g., labour cost).
Do you agree or disagree?

strongly disagree	disagree	mildly disagree	neutral	mildly agree	agree	strongly agree
☐	☐	☐	☐	☐	☐	☐

Q4.3. There is sufficient funding for change management (e.g., employment of an E-Health coordinator who is familiar with E-Health applications and can transfer knowledge to users).
Do you agree or disagree?

strongly disagree	disagree	mildly disagree	neutral	mildly agree	agree	strongly agree
☐	☐	☐	☐	☐	☐	☐

Q4.4. There is sufficient funding for employment of more staff due to a temporary loss of productivity at the early implementation stage.
Do you agree or disagree?

strongly disagree	disagree	mildly disagree	neutral	mildly agree	agree	strongly agree
☐	☐	☐	☐	☐	☐	☐

Q4.5. There is sufficient funding for technical support and system maintenance.
Do you agree or disagree?

strongly disagree	disagree	mildly disagree	neutral	mildly agree	agree	strongly agree
☐	☐	☐	☐	☐	☐	☐

Q5. Does the socio-cultural environment of the hospital support the new E-Health implementation?

Q5.1. Champions who are enthusiastic promoters of an innovation are always available for organisational innovation.
Do you agree or disagree?

strongly disagree	disagree	mildly disagree	neutral	mildly agree	agree	strongly agree
☐	☐	☐	☐	☐	☐	☐

Q5.2. There is always cooperation between departments of interest.
Do you agree or disagree?

strongly disagree	disagree	mildly disagree	neutral	mildly agree	agree	strongly agree
☐	☐	☐	☐	☐	☐	☐

Q5.3. The hospital is able to form partnerships.
Do you agree or disagree?

strongly disagree	disagree	mildly disagree	neutral	mildly agree	agree	strongly agree
☐	☐	☐	☐	☐	☐	☐

Q5.4. The hospital has got a variety of partnerships with other organisations.
Do you agree or disagree?

strongly disagree	disagree	mildly disagree	neutral	mildly agree	agree	strongly agree
☐	☐	☐	☐	☐	☐	☐

Q5.5. Partnerships for the new E-Health implementation, e.g., IT suppliers, are already available.
Do you agree or disagree?

strongly disagree	disagree	mildly disagree	neutral	mildly agree	agree	strongly agree
☐	☐	☐	☐	☐	☐	☐

Q5.6. Communication with other organisations for collaboration is efficient.
Do you agree or disagree?

strongly disagree	disagree	mildly disagree	neutral	mildly agree	agree	strongly agree
☐	☐	☐	☐	☐	☐	☐
